# A man with a worrying potassium deficiency

**DOI:** 10.1530/EDM-13-0067

**Published:** 2014-02-01

**Authors:** A Tabasum, C Shute, D Datta, L George

**Affiliations:** 1 Diabetes and EndocrinologyCardiff and Vale NHS TrustPenlan Road, Penarth, CF64 2XX, Cardiff, CF14 4BGUK; 2 BiochemistryCardiff and Vale NHS TrustPenlan Road, Penarth, Cardiff CF64 2XXUK

## Abstract

Hypokalaemia may present as muscle cramps and Cardiac arrhythmias. This is a condition commonly encountered by endocrinologists and general physicians alike. Herein, we report the case of a 43-year-old gentleman admitted with hypokalaemia, who following subsequent investigations was found to have Gitelman's syndrome (GS). This rare, inherited, autosomal recessive renal tubular disorder is associated with genetic mutations in the thiazide-sensitive sodium chloride co-transporter and magnesium channels in the distal convoluted tubule. Patients with GS typically presents at an older age, and a spectrum of clinical presentations exists, from being asymptomatic to predominant muscular symptoms. Clinical suspicion should be raised in those with hypokalaemic metabolic alkalosis associated with hypomagnesaemia. Treatment of GS consists of long-term potassium and magnesium salt replacement. In general, the long-term prognosis in terms of preserved renal function and life expectancy is excellent. Herein, we discuss the biochemical imbalance in the aetiology of GS, and the case report highlights the need for further investigations in patients with recurrent hypokalaemic episodes.

**Learning points:**

- Recurrent hypokalaemia with no obvious cause warrants investigation for hereditary renal tubulopathies.
- GS is the most common inherited renal tubulopathy with a prevalence of 25 per million people.
- GS typically presents at an older age and clinical suspicion should be raised in those with hypokalaemic metabolic alkalosis associated with hypomagnesaemia.
- Confirmation of diagnosis is by molecular analysis for mutation in the SLC12A3 gene.

## Background

Hereditary tubulopathies can present in adults with symptoms of recurrent hypokalaemia. They can present later in life due to a missed diagnosis at a younger age or patients being asymptomatic. Herein, we present a case of Gitelman's syndrome (GS) in an adult Romanian gentleman who had symptoms of recurrent hypokalaemia since childhood.

## Case presentation

A 43-year-old Romanian man was admitted to our hospital with excessive thirst and generalized weakness with a history of collapse a week before his admission. The collapse was characterized by a feeling of extreme tiredness and palpitations, with a fast irregular pulse, after a day of heavy physical labour and poor oral intake. The patient denied having a history of diarrhoea, vomiting, laxative or diuretic abuse, liquorice ingestion and excess alcohol intake.

Since childhood he had been suffering from intermittent muscular cramps, paraesthesia, excessive thirst, fatigue and carpopedal spasm. He had attributed these symptoms to his arduous work. At the age of 14 years, he was admitted to a local Romanian hospital following an episode of tetany and treated with an i.v. calcium (Ca+) infusion. As a child, he was noted to be short in comparison with his peers, but otherwise his development was unremarkable. His three siblings were all in good health.

Physical examination at the time of admission revealed a BMI of 24, and his vital observations were as follows: heart rate – 80/min, blood pressure – 130/88 mmHg, respiratory rate – 14 breaths/min, and oxygen saturation – 97% on air. Systemic examination proved to be unremarkable.

## Investigation

His ECG showed evidence of sinus arrhythmia, ST-segment depression in leads II, III and V4–V6, with a prolonged corrected QT interval (452 ms).

Biochemical analysis revealed low serum potassium levels of 2.3 mmol/l (3.5–5.3 mmol/l), magnesium levels of 0.58 mmol/l (0.7–1.0 mmol/l) and chloride levels of 94 mmol/l (95–108 mmol/l). High serum bicarbonate levels of 34 mmol/l (22–28 mmol/l), evidence of alkalosis, with a blood pH 7.59, and an elevated renin:aldosterone ratio were observed. Serum calcium, sodium, urea, creatinine, plasma glucose, thyroid function and serum cortisol tests were within the normal limits.

Urinalysis revealed urinary calcium levels to be abnormal at <0.5 mmol/l (1.25–3.75 mmol/l). Urinary potassium and chloride levels were high at 66 mmol/l (20–60 mmol/l) and 184 mmol/l (10–20 mmol/l) respectively.

Molecular analysis revealed a mutation in exons 9 and 16 of the *SLC12A3* gene, confirming a diagnosis of GS.

## Treatment and outcome

Treatment with oral and i.v. supplementation of potassium and magnesium was initiated during hospitalization of the patient. Serum potassium and magnesium levels improved gradually, but remained below the normal range. The patient was subsequently reviewed by a clinical biochemist who put him on potassium chloride, magnesium glycerophosphate and spironolactone treatment, which improved the corresponding levels significantly, but the levels remained borderline low. Despite this, the patient has much improved symptomatically since commencing the aforementioned treatment.

## Follow-up

He is currently being followed up as an outpatient and reviewed for bilateral knee pain secondary to chondrocalcinosis, confirmed by radiological imaging.

In view of the patient's family being in Romania, genetic counselling was not possible.

## Discussion

Our patient's clinical history was suggestive of chronic symptomatic hypokalaemia, which had remained undiagnosed for many years. Biochemical analysis revealed hypokalaemic hypochloraemic metabolic alkalosis with hypomagnesaemia and hypocalciuria suggestive of GS. Molecular analysis revealed a mutation in the thiazide-sensitive sodium chloride co-transporter expressed in the distal convoluted tubule (DCT). Bilateral chondrocalcinosis was secondary to the deposition of calcium pyrophosphate crystals induced by hypomagnesaemia.

GS is an autosomal recessive disorder caused by an inactivating mutation in the *SCL12A3* gene (chromosome 16q13), resulting in the loss of function of the sodium chloride co-transporter in the DCT. This mutation accounts for the majority of cases of GS (1). In a minority of patients, mutations in the *CLCNKB* gene, which encodes the chloride channel ClC-Kb are responsible (2). Other genes expressing proteins that promote distal tubular Mg transport include the cation channel subfamily 6 of the protein Claudin 16 (transient receptor potential channel subfamily M, member 6 (*TRPM6*)) and a recently identified epidermal growth factor, which acts as a distal regulator of TRPM6 activity (3). Mutations in these genes interfere with TRMP6 activity with consequent urinary Mg wasting and hypomagnesaemia (4). Studies have suggested that phenotypic variation in individuals with GS may be the result of mutations in different genetic loci (3).

### Electrolyte abnormality

The pathophysiology of hypokalaemic metabolic alkalosis is due to impaired functioning of the thiazide-sensitive NaCl co-transporter (NCCT) in the DCT. This results in solute loss causing volume depletion, which in turn stimulates renin/aldosterone secretion and juxtaglomerular hyperplasia, leading to hyperreninaemic hyperaldosteronism. This enhances potassium and hydrogen secretion in the collecting duct and results in the hypokalaemic metabolic alkalosis evident in GS.

Magnesium ions (Mg^2^^+^) are easily filtered through glomeruli after absorption from the small intestine. The majority of filtered Mg^2^^+^ are reabsorbed via passive paracellular diffusion: 50–70% of the ions are reabsorbed in the ascending limb of the loop of Henle and 10% in the proximal convoluted tubule (PCT). Finally, there is distal active transcellular diffusion, which depends on TRPM6 Mg channels. This plays an important role in urinary electrolyte excretion. Therefore, plasma Mg^2^^+^ levels depend on a balance between intestinal absorption and renal excretion (1).

The mechanism of hypomagnesaemia in GS and its relationship with normocalcaemic hypocalciuria are complex and still not well understood. Several mechanisms have been described, one of which postulates that genetic mutations that result in the reduced expression of TRPM6 Mg channels in the duodenum and DCT result in intestinal and urinary magnesium loss and hypomagnesaemia (5).

Reduced expression of the NCCT was described by Loffing *et al*. (5) in their experimental study on mice. They concluded that acute intratubular hydrochlorthiazide (HCTZ) infusion decreases the expression of the apical NCCTs with a consequent abrupt intracellular Ca+ passive diffusion that could induce distal convoluted cell (DCC) apoptosis. As a result, there is a reduction in the absorptive surface area in these tubular segments of the DCT, where the expression of TRPM channels regulates Mg^2^^+^ absorption.

In addition, inactivation of the NCCT might be responsible for reduced distal magnesium transport as well as up-regulation of calcium absorption in the DCT resulting in hypocalciuria (6).

Hypocalciuria in GS is also explained by volume depletion, which stimulates the sympathetic nervous system causing sodium and water reabsorption in the PCT. This creates a positive electrostatic intraluminal potential resulting in proximal paracellular Ca+ absorption (7).

The reason for normocalcaemia in GS is hypomagnesaemia itself, which induces the down-regulation of the calciotropic hormones parathyroid hormone and vitamin D, which in turn reduce skeletal sensitivity and intestinal calcium transport, hence leading to normocalcaemia (1).

Chondrocalcinosis occurs as a result of impaired pyrophosphatase enzyme activity, which enhances the crystallization of calcium pyrophosphate dehydrate deposited in the synovium and synovial fluid. This is induced by hypomagnesaemia (7).

GS is an autosomal recessive disorder with an estimated prevalence of ∼25 per million people, therefore, making it one of the commonest inherited renal tubular disorders (8). The condition typically presents in young adults. Individuals may be completely asymptomatic with electrolyte disturbance identified by routine biochemical analysis. Patients frequently report symptoms of fatigue, muscle weakness, facial paraesthesia and cramps (7). Our patient reported having had the above-mentioned symptoms since childhood, which was noticed particularly after heavy physical work during which additional electrolyte losses due to sweating occur. He did not seek medical attention as he attributed his symptoms to his manual work.

In cases of profound hypokalaemia and hypomagnesaemia, severe neuromuscular symptoms of tetany and paralysis may ensue and are particularly evident following episodes of diarrhoea or vomiting where there are additional electrolyte losses (8). Our patient was treated with a calcium infusion for tetany at the age of 14 years; however, he could not recollect a precipitating factor.

Cardiac arrhythmias may also be evident. Patients frequently report a history of palpitations, and ∼50% of patients will have a mild to moderate prolonged QT interval (3). Arterial hypotension may also be evident due to urinary salt wasting with resultant fluid loss (9). This was noticed by our patient, who reported of having collapsed a week before his admission. The collapse was characterized by a feeling of extreme tiredness and palpitations with a fast irregular pulse after a day of heavy physical work and poor oral intake. In a more detailed history regarding his dietary habits, he reported that he ate bananas on a daily basis. We conclude that this explains his mild and infrequent symptoms and his delayed presentation, as he was supplementing himself with potassium in the form of bananas. On the day of collapse, which is likely to be secondary to a cardiac arrhythmia, his oral intake was poor and he had been working heavily. A combination of increased electrolyte loss through sweating and reduced supplementary intake could have triggered this arrhythmia. His ECG on admission showed evidence of sinus arrhythmia, ST-segment depression in leads II, III and V4–V6, with a prolonged corrected QT interval (452 ms). These changes were in keeping with hypokalaemia and hypomagnesaemia. A prolonged QT interval is a risk factor for cardiac arrhythmias. ECG changes resolved after treatment with potassium and magnesium supplements.

Cardinal biochemical abnormalities that account for the aforementioned clinical symptoms include hypokalaemia, hypomagnesaemia, hypocalciuria and metabolic alkalosis.

### Differential diagnosis

GS should be suspected in patients with hypokalaemic metabolic alkalosis that cannot be explained by another obvious cause such as diarrhoea, vomiting, laxative and diuretic abuse, liquorice ingestion or excess alcohol intake. It is differentiated from diuretic abuse by careful history and screening for urinary diuretics. Laxative abuse causes metabolic acidosis with a urine potassium:creatinine ratio <1.5.

Certain endocrine disorders, for example, primary hyperaldosteronism, congenital adrenal hyperplasia and Cushing's syndrome, can present with a similar biochemical picture associated with hypertension. The above-mentioned conditions can be excluded by low serum magnesium, high cortisol and high aldosterone/renin levels and response to spironolactone.

Another condition to be borne in mind as a differential diagnosis in GS is thyrotoxic periodic paralysis (TTP) featured by attacks of muscle paralysis in the presence of hyperthyroidism, and hypokalaemia is usually present during attacks. Our patient's thyroid function test was within the normal limit, so TTP was excluded.

A comparison of GS with the other renal tubulopathies, Bartter and Liddle's syndromes, is given in Table 1.

**Table 1:** Differentiating features of three most common hereditary tubulopathies

**Features**	**Gitelman's syndrome**	**Bartter syndrome**	**Liddle's syndrome**
Inheritance	Autosomal recessive	Autosomal recessive	Autosomal dominant
Mutation	*SLC12A3* (NCCT) and *CLCNKB*	*SLC12A1*, *CLCN1CB* and *KCNJ1*	*SCNN1B* or *SCNN1G*
Age at presentation	Late childhood/adulthood	Neonatal stage/infancy/adulthood	Childhood/adulthood
Symptoms	Non-specific/musculoskeletal/chondrocalcinosis	Polyuria/polydipsia/failure to thrive/seizure/tetany	Non-specific/weakness/fatigue/symptoms of hypertension
Biochemical	Hypochloraemic hypokalaemic alkalosis	Hypochloraemic hypokalaemic alkalosis	Hypernatraemic, hypokalaemic alkalosis
	Hypomagnesaemia	Increased renin:aldosterone ratio	Low rennin:aldosterone ratio
	Increased renin:aldosterone ratio		
Urine analysis	Low calcium levels	High calcium/PGE_2_ levels	Normal calcium levels
Nephrocalcinosis	No	Yes	No
Treatment	K/Mg^2^^+^ replacement	K replacement and potassium-sparing diuretics	Low-salt diet
	Potassium-sparing diuretics	PG synthetase inhibitor	Amiloride

ENaC, epithelial sodium channel; PGE_2_, prostaglandin E_2_; K, potassium; Mg^2^^+^, magnesium; PG, prostaglandin.

### Treatment

A tubulopathy cannot be modified; therefore, GS is treated with long-term potassium and magnesium salt replacement. High doses of potassium chloride, up to 500 mEq/kg, are required to establish normal serum concentrations, but this can cause gastrointestinal intolerance. Therefore, the therapeutic goal is to maintain asymptomatic stable hypokalaemia (1).

Treatment of hypomagnesaemia with magnesium chloride, up to 4–5 mEq/kg in three to four divided doses, is better tolerated. In addition, the aldosterone antagonist spironolactone can be of benefit as it can counteract compensatory secondary hyperaldosteronism and help stabilize potassium levels. However, the use of spironolactone may result in hypovolaemia and hyponatraemia and hence appropriate salt and fluid intake should be advised.

These patients should be given advice on reduced alcohol intake and avoidance of liquorice, iced tea and lemon juice, as these can exaggerate electrolyte disturbance. In addition, these patients should be encouraged to take potassium-rich foods.

### Prognosis

In general, the long-term prognosis in terms of preserved renal function and life expectancy is excellent if treated early and adequately. However, these patients are at a risk of cardiac arrhythmias due to severe hypokalaemia and hypomagnesaemia (10).

Scognamiglio *et al*. (11) showed that physical exercise may induce coronary microvascular and myocardial defects in GS patients. This would warrant detailed cardiac assessment in GS patients. They should be discouraged to participate in sporting activities, as this can trigger malignant arrhythmias. Muscular symptoms can interfere with physical activity and hence quality of life.

### Genetic counselling

Genetic counselling is important in GS. As it is inherited in an autosomal recessive pattern, there is a recurrence risk of 25% for parents with an affected child. If the parents who already have other child(ren) who are clinically well, it is not absolutely certain that they will not have the disease, as it can appear later in life. If the parents are eager to know the status of other child(ren), DNA analysis can be carried out in the other child(ren). An affected adult patient has a low risk of having children with GS (∼1 in 400), unless the patient's partner is consanguineous. Antenatal diagnosis of GS is not advised as it carriers a good prognosis in a majority of patients.

## Conclusion

There should be a high index of suspicion for hereditary tubulopathies in patients presenting with recurrent hypokalaemia and no obvious cause has been identified. GS can present later in life, even in manual workers with excessive sweating, as they can counterbalance the hypokalaemia with increased dietary supplementation.

## Funding statement

This case report did not receive any specific grant from any funding agency in the public, commercial or not-for-profit sector. The authors did not receive any funding from any organization.

## Patient consent

Informed written consent was obtained from the patient for the publication of this case report.

## Author contribution statement

A Tabasum was responsible for writing the manuscript, researching the discussion, and reviewing and editing the manuscript; C Shute wrote, reviewed and edited the manuscript; D Datta helped in biochemical and genetic analyses; and L George is the senior author and was responsible for supervision, obtaining patient's consent, and reviewing and editing the final manuscript.
